# CRYSTALLBrain: crystalloid fluid choice and neurological outcome in patients with non-traumatic subarachnoid haemorrhage—a study protocol for a multi-centre randomised double-blind clinical trial

**DOI:** 10.1186/s13063-025-09099-9

**Published:** 2025-10-17

**Authors:** Anna S. Messmer, Matthieu Pitteloud, Hervé Quintard, Urs Pietsch, Martin Müller, Miodrag Filipovic, Stephan M. Jakob, Werner J. Z’Graggen, Joerg C. Schefold, Carmen A. Pfortmueller

**Affiliations:** 1 Department of Intensive Care Medicine, Inselspital, Bern University Hospital and University of Bern, Bern, Switzerland; 2 Department of Intensive Care, Neuro-Intensive Care Unit, University Hospital of Geneva, Geneva, Switzerland; 3 Department of Perioperative Intensive Care Medicine, Cantonal Hospital St. Gallen, St. Gallen, Switzerland; 4 Department of Emergency Medicine, Inselspital, Bern University Hospital and University of Bern, Bern, Switzerland; 5 University of Bern, Bern, Switzerland; 6 Department of Neurosurgery, Inselspital, Bern University Hospital and University of Bern, Bern, Switzerland

**Keywords:** Subarachnoid haemorrhage, Crystalloid fluid choice, Vasospasms, Outcome, Neurological sequelae

## Abstract

**Background:**

Vasospasms are common in patients presenting with non-traumatic subarachnoid haemorrhage (SAH) and are the main contributor to long-term disability or death in these patients. The key immediate management of vasospasms is the improvement of brain perfusion by the administration of intravenous fluid and vasopressors if needed. Yet, there is no clear recommendation regarding the choice of fluid in this particular patient population. Data suggests a survival benefit using normal saline in patients with TBI; however, its impact on outcomes in patients with SAH is lacking. Thus, the aim of this study is to evaluate whether the use of normal saline reduces clinically relevant vasospasms compared to Ringer’s lactate in patients with SAH.

**Methods:**

Patients presenting with non-traumatic SAH will be randomised 1:1 to normal saline or Ringer’s lactate group. Blinded study fluid will be used exclusively for resuscitation and maintenance until ICU/IMC discharge or a maximum of 14 days, whichever occurs first. Management of vasospasms and general management of the SAH patient will be according to the clinic standard of care. Primary endpoint is the occurrence of clinically relevant vasospasms. Key secondary outcomes include mortality, severity and treatment of vasospasms, and neurological outcomes at 90 days.

**Discussion:**

The proposed randomised controlled trial offers a safe, non-invasive way to gain insights about crystalloid fluid choice in SAH patients, with potential to improve outcomes in this critically ill patient group. This study could establish a new gold standard in fluid therapy for neuro-critical care.

**Trial registration:**

The trial is registered on ClinicalTrials.gov (date of registration 18 June 2021) and on the Swiss National Clinical Trials Portal, SNCTP000004575.

**Supplementary Information:**

The online version contains supplementary material available at 10.1186/s13063-025-09099-9.

## Administrative information

Note: the numbers in curly brackets in this protocol refer to SPIRIT checklist item numbers. The order of the items has been modified to group similar items (see http://www.equator-network.org/reporting-guidelines/spirit-2013-statement-defining-standard-protocol-items-for-clinical-trials/ ).

**Table:** 

Title {1}	Crystalloid fluid choice and neurological outcome in patients with non-traumatic subarachnoid haemorrhage (CrystallBrain)—a multi-centre randomised double-blind clinical trial
Trial registration {2a and 2b}	ClinicalTrials.gov, NCT04043598 Swiss National Clinical Trials Portal, SNCTP000004575
Protocol version {3}	10.05.2024 V 2.2
Funding {4}	Departement für Lehre und Forschung, Bern University Hospital and University of Bern, Bern, Switzerland OPO Foundation (Grant 2020-0013) Anaesthesiology and Intensive Care Foundation (Stiftung für Forschung in der Anästhesie und Intensivmedizin (Grant 33/2020)
Author details {5a}	Anna S. Messmer^1^, Matthieu Pitteloud^1^, Hervé Quintard^2^, Urs Pietsch^3,4^, Martin Müller^4^, Stephan M. Jakob^5^, Werner J. Z’Graggen^6^, Joerg C. Schefold^1^, Carmen A. Pfortmueller MD^1^ ^1^Department of Intensive Care Medicine, Inselspital, Bern University Hospital and University of Bern, Bern, Switzerland ^2^Department of Intensive Care, Neuro-Intensive Care Unit, University Hospital of Geneva, Geneva, Switzerland ^3^Department of perioperative Intensive Care Medicine, Cantonal Hospital St. Gallen, St. Gallen, Switzerland ^4^Department of Emergency Medicine, Inselspital, Bern University Hospital and University of Bern, Bern, Switzerland ^5^University of Bern, Bern, Switzerland ^6^Department of Neurosurgery, Inselspital, Bern University Hospital and University of Bern, Bern, Switzerland
Name and contact information for the trial sponsor {5b}	Prof. Dr.med. Carmen A. Pfortmueller Department of Intensive Care Medicine Inselspital, Bern University Hospital and University of Bern Freiburgstrasse 10 3010 Bern, Switzerland
Role of sponsor {5c}	Investigator-initiated trial

## Introduction

### Background and rationale {6a}

Non-traumatic subarachnoid haemorrhage (SAH) is a devastating disease with a high mortality of up to 50% [1]. More than 50% of the survivors develop cognitive dysfunctions in the long term and never return to their previous status [1]. Most of these patients are Younger than 55, so the disease has a major socio-economic impact in addition to the individual tragedy [1, 2].

Despite the advances in diagnosis and treatment of SAH, effective therapeutic interventions are still limited and clinical outcomes remain disappointing [1]. In addition to the early brain injury caused by the haemorrhage and inflammatory response to it, about one third of the patients with SAH develop delayed cerebral ischaemia (DCI) [3]. DCI is a primary contributor to persistent neurological impairment in patients with SAH [4–6]. DCI often results from vasospasms of proximal cerebral arteries and the microcirculation; however, there might be also other factors contributing to impaired cerebral perfusion and subsequently ischaemic injury, such as seizures, impaired autoregulation with reduction of regional blood flow, microthrombosis, spreading depolarisations, or high intracranial pressures [7]. Yet, approximately half of the patients with angiographic vasospasms progress to develop DCI, and spasms in small vessels may not be detected through standard angiography [5]. The pathophysiology underlying vasospasms, a primary driver of secondary brain injuries, remains incompletely understood; however, evidence suggests that oxidative stress, inflammation in response to haemorrhage, decrease of NO as well as episodes of hyponatraemia contribute to alterations in cerebral arterial diameter [8]. The latter is a common phenomenon in patients with SAH and occurs in 30 to 50% of the patients [9]. Although the only guideline-recommended strategies for reducing DCI include enteral nimodipine administration and the prevention of hypovolaemia and hypotension, primarily through fluid administration, there are currently no specific recommendations regarding the choice of crystalloid fluids for this patient population [10, 11]. Of note, patients with SAH often require several litres of intravenous fluid per day in order to counterbalance overt fluid losses and maintain adequate blood pressure. In a recent meta-analysis, Zampieri and colleagues summarise the current evidence on balanced versus normal saline infusion in critical care [12]. The study indicates a potential survival benefit associated with the use of balanced fluids in the general critical care population [12]. However, the meta-analysis also reveals that the administration of balanced fluids in patients with traumatic brain injury is associated with increased mortality [12]. This finding is consistent with other research suggesting that neuro-intensive care patients may benefit from a higher sodium load [3, 8, 11–13]. Similar might be true for patients with non-traumatic SAH. However, according to the guidelines by the ESICM, no prospective randomised trial has ever evaluated whether the type of infusion fluid used in patients with SAH might influence the incidence of vasospasms, and mortality and morbidity in these patients, and further investigations are highly warranted [11].

### Objectives {7}

#### Hypothesis

We hypothesise that the use of a sodium-enriched solution (0.9% saline) for fluid therapy in patients with non-traumatic SAH will result in a lesser occurrence of clinically significant vasospasm requiring immediate intervention than after treatment with a balanced, formally hypotonic infusion solution (Ringer’s lactate).

#### Primary objective

The primary aim of this study is to evaluate whether the use of normal saline results in lower occurrence of clinically relevant vasospasms when compared to lactated Ringer’s in patients with non-traumatic SAH.

### Trial design {8}

The CrystallBrain study is an investigator-initiated randomised controlled multi-centre double-blind trial comparing 0.9% saline to lactated Ringer’s (two already established treatment protocols) in patients with non-traumatic SAH. Patients will be allocated a 1:1 ratio to either normal saline (0.9%) or lactated Ringer’s.

## Methods: participants, interventions, and outcomes

### Study setting {9}

The study will be carried out in accordance with the protocol and with principles enunciated in the current version of the Declaration of Helsinki, the guidelines of Good Clinical Practice (GCP) issued by ICH [13, 14]. We plan to enrol 320 patients (1:1 randomisation) at Intensive Care Units at the University Hospital Bern, Inselspital, Bern, at the University Hospital Geneva, HUG, Geneva, and at the Canton Hospital St. Gallen.

### Eligibility criteria {10}

#### Inclusion criteria

All adult patients suffering from non-traumatic SAH and admitted to the Intensive Care Unit (ICU) or Intermediate Care Unit of one of the study centres are eligible for inclusion.

#### Exclusion criteria

- Patient with major intracranial trauma
- Diagnosis of an AV-malformation as the source of SAH on the primary CT/MRI or angiography if performed prior to randomisation
-  > 24 h since first diagnosis of SAH (as diagnosed by cerebral imaging [CT or MRI])
- Patients with clear limitation to therapy at hospital admission (e.g. ICU admission for evaluation of organ donation)

### Who will take informed consent? {26a}

The process leading to informed consent will be in compliance with applicable regulations and national laws. The informed consent procedure will be as follows: independent physician signature will be sought prior to enrolment of the patient. If the patient presents with Glasgow Coma Scale (GCS) 15, then patient consent will be reached prior to randomisation. If the patient is cognitively impaired (GCS < 15), consent of the patient’s next of kin will be sought, with deferred informed consent by the patient. The investigators will explain to each participant or next of kin the nature of the study, its purpose, the procedures involved, the expected duration, the potential risks and benefits, and any discomfort it may entail.

### Additional consent provisions for collection and use of participant data and biological specimens {26b}

No additional biological specimens will be collected and stored. Informed consent for the potential future use of trial data has been obtained from all participants and is documented in the consent form.

## Interventions

### Explanation for the choice of comparators {6b}

Worldwide, 0.9% sodium chloride (normal saline) is the most commonly administered fluid in critical care [15–18]. In contrast to other infusion solutions like lactated Ringer’s, normal saline does not contain other electrolytes than sodium and chloride and is therefore considered a non-buffered solution. Due to the high chloride content, there were rising concerns about the development of metabolic acidosis, kidney injury, and even higher mortality in patients receiving normal saline [17–22]. This in turn has led to the increased use of buffered solutions, such as Ringer’s lactate.

In contrast to isotonic saline, it contains calcium, potassium, magnesium, and the metabolisable anion lactate. Lactate is metabolised to bicarbonate (also referred to as “buffer concept”). This is important for two reasons: First, through infusion of lactate containing solutes, the bicarbonate concentration of the plasma remains stable and is not diluted [23, 24]. Secondly, the use of a metabolisable anion such as lactate saves the use of other anions such as chloride and avoids hyperchloraemia. Studies showed that in comparison to isotonic saline, the occurrence of metabolic acidosis and electrolyte abnormalities is significantly reduced when a balanced infusate is used [25–36]. A recent meta-analysis has revealed that the use of buffered infusion solutions in the critically ill leads to a survival benefit, with the exception of patients with traumatic brain injury, where buffered solutions with lower salt content were associated with higher mortality [12]. However, the impact of fluid choice in patients with non-traumatic SAH is not known. To date, both infusates are used as standard solutions in the treatment of patients with SAH in ICU worldwide.

### Intervention description {11a}

All patients that meet the eligibility criteria as defined above will be randomised 1:1 to either the normal saline group or the Ringer’s lactate group. We will use 0.9% saline and Ringer’s lactate (Bichsel©, Interlaken, Switzerland). Study fluids will be used exclusively for resuscitation and maintenance until ICU/IMC discharge or a maximum of 14 days, whichever occurs first.

Fluid will generally be administered according to haemodynamic and clinical endpoints following standard of care of the participating centres. Patients will receive the allocated study fluid for all fluid therapy during the study period. All other fluid types including colloids will not be administered during the study time with the exception of creep fluid necessary to administer medication. In addition, hypertonic saline perfusion is allowed to treat low sodium levels and for treatment of brain oedema. Mannitol is allowed for the treatment of acute elevation of intracranial pressures and/or brain oedema. During interventions or surgery, continuous treatment with the allocated study fluid will be attempted.

#### Management of vasospasm

All patients with SAH will receive prophylactic treatment with nimodipine if blood pressure allows. Vasospasms are either diagnosed clinically according to the CONCIOUS criteria [6] or radiologically by cerebral Doppler examination, cerebral CT-angiography, and/or CT perfusion imaging. If vasospasms are clinically significant, immediate treatment entails. The augmentation of the mean arterial pressure as the first step of management. The target is set according to the patient’s clinical presentation and is at the discretion of the treating team (intensivist, neurosurgeon). Fluid administration with the allocated fluid type will be the first-line therapy to augment blood pressure, followed by a norepinephrine infusion. If the vasospasms are unresponsive to conventional therapy, the patient will undergo endovascular treatment. The choice of treatment (intra-arterial application of vasodilators or angioplasty) is at the decision of the treating neurosurgeon, interventional neuroradiologist, and/or intensivist.

#### Management of electrolyte disorders

In the case of the occurrence of hypo- or hypernatraemia, concomitant measurement of serum and urine osmolality and urine sodium will be performed to evaluate the nature of the sodium disorder (e.g. diabetes insipidus, syndrome of inappropriate antidiuretic hormone secretion) and treatment will be according to the diagnosis and the clinical situation of the patient following the clinics SOPs. Study fluids will be continued as allocated.

### Criteria for discontinuing or modifying allocated interventions {11b}

The clinical team may at any time violate the protocol if they find it to be in the best interest of the participant. The procedure of handling withdrawal of consent from a participant will follow national regulations. Patients diagnosed with an AV-malformation (AVM) as the source of the non-traumatic SAH after the randomisation (i.e. the AVM was not evident in the initial CT or MRI done prior to randomisation) will drop out of the study and the data will be anonymised for a drop-out analysis.

### Strategies to improve adherence to interventions {11c}

All health care providers on the ICUs will be trained to prescribe only the study fluids in all included patients. In addition, patients and their family members will receive comprehensive information with the consent procedure including the importance of completing the follow-up. Furthermore, they receive contact details of the respective study team in case questions or concerns should arise.

Except for the choice of fluid given, all other treatment required will be provided within the standard of care for all patients. Protocol deviations will be recorded in the database.

### Relevant concomitant care permitted or prohibited during the trial {11d}

All concomitant treatments are permitted during the study, with the exception of the administration of maintenance or resuscitation fluids other than the blinded study fluid (e.g. normal saline or Ringer’s lactate solution, colloids, dextrose).

### Provisions for post-trial care {30}

As this study is a comparison of two already established fluid protocols, no harm is anticipated.

### Outcomes {12}

#### Primary endpoint

The primary endpoint is the occurrence of clinically relevant vasospasm requiring immediate intervention as mentioned above during ICU/IMC stay. Clinically relevant vasospasm will be defined as any vasospasm requiring medical intervention (conservative or endovascular). The outcome will be analysed as a dichotomous variable.

#### Secondary endpoints

Secondary endpoints entail ICU and in-hospital mortality and 90-day mortality (aggregated as proportions of patients), treatment modalities for vasospasms (conservative vs. endovascular treatment—proportion of patients, number of days), need for decompressive hemicraniectomy (proportion of patients), evidence of brain oedema requiring intervention (proportion of patients), occurrence of hyper- or hyponatraemia (proportion of patients and number of days with hyper- or hyponatraemia), length of ICU and hospital stay (days), and neurological outcome at day 90 (ordinal scale) (see also Table 1).

**Table 1:** Outcomes

Primary endpoint	Occurrence of clinically relevant vasospasm requiring immediate intervention during ICU/IMC stay
Secondary endpoints	
Mortality	ICU- and in-hospital mortality 90-day mortality
Length of stay	Length of ICU/IMC stay Length of hospital stay
Treatment modalities for vasospasms	Vasospasms requiring endovascular treatment Vasospasms managed conservatively
Brain oedema	Requiring any of the following: - Deep sedation - Ventricular drainage - Hyperventilation - Osmotherapy - Decompressive craniectomy
Electrolytes	Occurrence of hyponatraemia Occurrence of hypernatraemia
Neurological outcome at 90 days	Assessed by: - Modified Rankin Scale - Glasgow Outcome Scale Extended - Mini Montreal Cognitive Assessment

*ICU *Intensive Care Unit, *IMC *Intermediate Care Unit

#### Pre-planned substudies

In pre-planned substudies, we aim to analyse the following: (1) electrolyte shifts over the course of the duration of the active study phase, (2) fluid accumulation during ICU or IMC stay, (3) neuro-cardiac interactions, and (4) blood pressure dynamics in the context of vasospasms.

### Participant timeline {13}

All eligible patients will be randomised within 24 h of diagnosis (time stamp on first computed tomography or MRI). From randomisation up to discharge from ICU/IMC or day 14 after randomisation, whichever comes first, the patient will receive the allocated study fluids only. Fluid, water, and electrolyte balance will be assessed daily. Serum osmolality and urinary sampling will be performed at hospital admission (day 1) and on days 3, 7, 10, at day 14, or at the day of ICU/IMC discharge, whichever comes first. In addition, blood gas analysis including electrolytes will be performed at a minimum of once a day as long as the patient is in ICU/IMC. In addition, an electrocardiogram and, at the lead centre (University Hospital Bern, Inselspital), bioimpedance measures will be performed on predefined study days (see Table 2 [SPIRIT figure]).

**Table 2:**
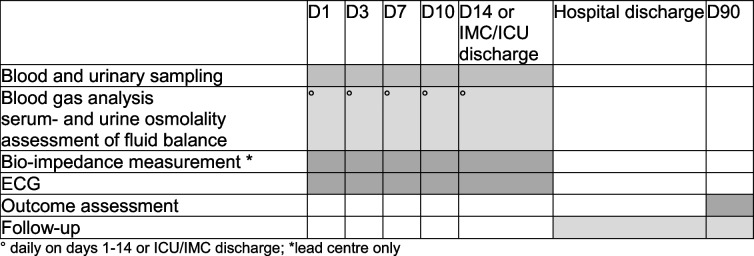
SPIRIT figure

Neurological outcome will be assessed by the modified Rankin Scale (mRS), Glasgow Outcome Scale Extended (GOSE), and Montreal Cognitive Assessment (miniMOCA) at day 90 (see Supplementary Figures S1–S3). Mortality will be assessed at day 30 and 90.

### Sample size {14}

Sample size calculation was based upon our ICU’s registry data (PDMS). In 2017, 71/160 (44.38%) patients with SAH reached our predefined endpoint of clinically relevant vasospasm requiring immediate intervention. Studies evaluating other prophylactic measures against vasospasm, such as clazosentan, magnesium, or cilostazol, have reported relative risk reductions ranging from approximately 10% to 50% [37]. Based on these findings, we considered the expected effect of the fluid intervention to lie in the mid-range, corresponding to about a 30% relative risk reduction. With an assumed baseline event rate of 45%, this translates into an absolute risk reduction of approximately 15 percentage points. The sample size was calculated with the Power and Sample Size Calculator provided by the Statistical Institute of the Medical University of Vienna (http://statistics.msi.meduniwien.ac.at) and cross checked on http://powerandsamplesize.com. The calculation revealed that at least 160 patients per group are required to detect a reduction of 15% of clinically relevant vasospasms with an alpha-error of 0.05, a power of 80%, and a drop-out rate of 15%.

### Recruitment {15}

All centres will perform daily screening of all ICU admissions in order to secure adequate recruitment. Recruitment of study participants will be reported to the trial sponsor and lead principal investigator via automated alert system in REDCap. Patients with SAH are critically ill and a vast majority of patients stay on the ICU or IMC for 14 days after the diagnosis; thus, loss of follow-up or other events are unlikely to interfere with the primary outcome. In addition, patients with SAH are treated in dedicated centres and—as part of their routine management—are being followed up by the neurosurgical team. Thus, we expect to gain information about their neurological outcome from a majority of our enrolled patients.

## Assignment of interventions: allocation

### Sequence generation {16a}, concealment mechanism {16b}, and implementation {16c}

REDCap will be used to allocate eligible patients in a 1:1 ratio to either the normal saline group or the lactated Ringer’s group. Randomisation will be stratified by trial site. Dedicated and trained study personnel at all sites will enrol patients and subsequently inform the team of the patients’ allocation. A box with the blinded study fluids will be given to the treating intensive care physician with the patient’s study number. Study fluid will be blinded so that no identification of the study fluid will be possible by the treatment team. The randomisation list will be held by the study’s data manager (member of the CTU of the University of Bern). All patient data will be encoded using a code for each patient included in the study. Decoding will only be possible for the primary investigator of the study. No data on patients’ identity will be presented.

## Assignment of interventions: blinding

### Who will be blinded {17a}

All study fluids are blinded by the manufacturer. All investigators, trial participants, care providers, outcome assessors, and data analysts will be blinded to patients’ allocation.

### Procedure for unblinding if needed {17b}

A list for emergency unblinding of the study will be held by research nurses of the Department of Anaesthesia, Inselspital, Bern. Access will be possible 24 h through the research nurse on call.

## Data collection and management

### Plans for assessment and collection of outcomes {18a} and plans to promote participant retention and complete follow-up {18b}, and data management {19}

Data will be obtained from the participant’s hospital files, study-specific examinations (e.g. bioimpedance results), the Insel Data Science Centre (IDSC) and national registries (mortality data) and by participant survey/interview and entered in the web-based eCRF (REDCap) by authorised trial personnel. For participants transferred from a trial ICU to a non-trial ICU, data related to the outcomes will be collected according to investigator contact with the non-trial ICU.

Long-term disability will be assessed by telephone interview or at the outpatient clinic appointment in the neurosurgical unit based on the modified the mRS, GOSE, and miniMOCA (see Figures S1–S3 in the Supplemental File for more details).

All data will be verified by regular monitoring, as well as data validity checks and automated consistency queries in the trial database. A final data quality check will be performed before the database is closed. All data changes and checks are tracked in the database.

### Confidentiality {27}

Each participant will receive a unique trial identification number. Trial investigators will receive a personal username and password to access the randomisation system and the eCRF. Each site will only have access to site-specific participant data. Data will be handled according to Swiss law. Study documentation and data are accessible to auditors/inspectors from the ethics committee and other regulatory authorities at any time. All parties involved will keep individual data strictly confidential.

### Plans for collection, laboratory evaluation, and storage of biological specimens for genetic or molecular analysis in this trial/future use {33}

No biological data will be stored.

## Statistical methods

### Statistical methods for primary and secondary outcomes {20a}

This is a parallel group study. All randomised patients receiving the study fluid will be included in the primary analysis according to a modified intention-to-treat (mITT) principle [38]. The analysis is defined as modified because patients in whom an AV-malformation is subsequently identified as the source of the haemorrhage will be excluded, since this underlying condition falls outside the intended study population; their data will contribute only to a drop-out analysis. Normal distribution of continuous variables will be tested with the Shapiro–Wilk test. Normally distributed variables are presented with mean and standard deviation. Skewed or interval and ordinal variables are presented as medians with interquartile ranges. Normally distributed interval and ordinal variables will be compared by unpaired *t*-test and skewed variables by the Wilcoxon rank sum test. Comparisons of categorical variables were performed using chi-square test or Fisher’s exact test, as appropriate [39].

Whether the use of normal saline significantly influences our primary endpoint will be assessed by a logistic regression analysis adjusted for the stratification variables (trial site) in the ITT population [40]. For the primary endpoint, deceased patients or patients who were transferred prior to reaching the primary endpoint will be counted as “no event”. A sensitivity analysis with a modified primary endpoint will be used, defined as a composite endpoint of (i) clinically relevant vasospasms during the first 14 days, (ii) ICU/IMC readmission during the 14 days (if discharged earlier) because of vasospasms, or (iii) death within 14 days.

Our secondary endpoints will be assessed by logistic (binary endpoints) as well as linear regression models (such as mRS, GOSE, and miniMOCA) adjusted for the stratification variable (trial site).

We will report absolute risk difference and relative risk ratios with 95% CIs for dichotomous secondary outcomes. For continuous secondary outcomes, we will report mean differences with 95% CIs. Missing values will be imputed with the use of the last-observation-carried-forward method for measurements made after baseline. *P* values smaller than 0.05 are considered statistically significant. Secondary outcomes will be analysed in an explorative way.

### Interim analyses {21b}

No interim analysis will be performed.

### Methods in analysis to handle protocol non-adherence and any statistical methods to handle missing data {20c}

A logistic regression analysis in the per protocol population adjusted for the stratification variable (trial site) is planned. Missing data is handled by the last observation carried forward method. In case the number of drop-outs exceeds the expected rate of 15%, the excess drop-outs will be replaced by recruitment of new subjects.

### Plans to give access to the full protocol, participant-level data, and statistical code {31c}

Protocol, dataset, and statistical code will be made available upon reasonable request.

## Trial oversight and monitoring

### Composition of the coordinating centre and trial steering committee {5d}

The CrystallBrain study is managed by the lead centre, University Hospital Bern, Inselspital Bern, Switzerland, and by local research teams at each study site. Prior to the start of the study, all research and clinical staff involved in the treatment of study patients will undergo formal training. All staff involved in executing trial tasks require proof of GCP training, and training will be logged in a dedicated form. Database programming support will be provided by the CTU of the University of Bern, Switzerland. Study monitoring is coordinated by the trial sponsor.

### Composition of the data monitoring committee, its role and reporting structure {21a}

As this is a comparison of two already established fluid protocols, this trial is categorised as low-risk study. Therefore, a data monitoring board was waived by the respective ethical committee.

### Adverse event reporting and harms {22}

Severe adverse events (SAEs) will be reported in a standardised fashion according to Swiss law.

All SAEs are documented and reported immediately (within a maximum of 24 h) to the sponsor-investigator of the study.

### Frequency and plans for auditing trial conduct {23}

There are no plans to conduct an independent audit of the trial conduct.

### Plans for communicating important protocol amendments to relevant parties (e.g. trial participants, ethical committees) {25}

Every new protocol amendment will be communicated with the lead and the respective local ethical committee, and approval will be sought.

### Dissemination plans {31a}

All trial results whether positive, negative, or neutral will be published preferably in a peer-reviewed medical journal. Authorship will be granted according to the guidelines from the International Committee for Medical Journal Editors (ICMJE; http:{www.icmje.org). The funding sources will be acknowledged, but they will have no influence on the data handling or analyses, the writing of the manuscript, or the decision to publish.

## Discussion

The proposed randomised controlled trial is a suitable and non-invasive way to gain further knowledge on the impact of crystalloid fluid choice on the outcome of patients with non-traumatic SAH. The management of these patients is complex and requires specialised centres to address both early and late complications of this serious condition [5]. Vasospasms, which can lead to delayed cerebral ischaemia, are a significant contributor to poor neurological outcomes in SAH patients. A primary focus in SAH management is maintaining adequate cerebral perfusion by preventing hypotension and hypovolaemia, often necessitating large volumes of intravenous fluids [41]. The European Society of Intensive Care Medicine (ESICM) recommends both balanced solutions and normal saline as safe and preferred first-line options for fluid resuscitation in neurocritically ill patients [11]. However, recent evidence suggests that the choice of crystalloid fluid influences mortality in critically ill patients, with balanced fluids generally benefiting most patients, except those with traumatic brain injury [12]. A potential explanation for the observed mortality difference may be the varying salt loads associated with different crystalloid solutions [42]. Recent evidence indicates that the high salt content in infusion solutions used in critical care plays a substantial role in fluid accumulation; the latter is associated with increased mortality in the general critically ill population [43, 44]. However, neurocritically ill patients, such as patients with traumatic brain injury or our population with SAH, are prone to developing hypo- or hypernatraemia due to their underlying neurological disease. Evidence suggests that low serum sodium levels may contribute to an increased risk of delayed cerebral injury in this patient population; therefore, maintaining stable sodium levels is essential in their management and may account for the observed beneficial effects on survival in patients with traumatic brain injury receiving normal saline [12]. Similar might be true for patients with SAH; however, to date, the evidence is lacking. A subgroup analysis of the SMART trial (Isotonic Solutions and Major Adverse Renal Events Trial), which compared balanced crystalloids with non-traumatic SAH, demonstrated no significant difference in 90-day mortality or neurological outcomes [41]. However, the analysis included only 79 patients and thus is underpowered. Furthermore, this study was unblinded, allowing clinicians discretion to administer non-assigned crystalloids if deemed necessary for patient care. In the SAH patient subgroup, there was a significantly higher rate of crossover compared to the overall trial, primarily affecting the balanced-crystalloid group, which may have potentially influenced the trial’s outcome results. Additionally, hyponatraemia management was not standardised in the SMART trial and was at the discretion of the treating physician.

The present study is to our knowledge the first blinded multi-centre randomised trial investigating the impact of crystalloid fluid choice in patients with non-traumatic SAH. We therefore believe this trial can result in well-grounded recommendations for fluid preferences in this patient population.

## Trial status

Protocol version 2.2, November 2024. Recruitment of the first participant was in May 2022 at the lead centre, University Hospital Bern, Inselspital, and in August 2022 at the University Hospital Geneva, HUG. The Canton Hospital St. Gallen is planning to start the trial in early 2025. The expected time of end of the trial from all sites is April 2026. To date, 157 (49%) patients have been randomised in the trial.

## Supplementary Information

Supplementary material 1.

## Data Availability

Data will be available upon reasonable request from the corresponding author.

## Abbreviations

AVM: Arterio-venous malformation
CT: Computed tomography
CTU: Clinical Trial Unit
eCRF: Electronic case report form
ESICM: European Society of Intensive Care Medicine
GCP: Good Clinical Practice
GCS: Glasgow Coma Scale
GOSE: Glasgow Outcome Scale Extended
HUG: Hopital Universitaire Geneve, University Hospital Geneva
ICMJE: International Committee for Medical Journal Editors
ICU: Intensive Care Unit
IDSC: Insel Data Science Centre
IMC: Intermediate Care Unit
ITT: Intention to treat
miniMOCA: Mini Montreal Cognitive Assessment
MRI: Magnetic resonance tomography
mRS: Modified Rankin Scale
PDMS: Patient data management system
SAH: Subarachnoid haemorrhage
SAP II: Simplified acute physiology score
SOFA: Sequential organ failure assessment
TBI: Traumatic brain injury
TISS-28: Therapeutic intervention scoring system
