# Supplementary material for: CRYSTALLBrain: crystalloid fluid choice and neurological outcome in patients with non-traumatic subarachnoid haemorrhage—a study protocol for a multi-centre randomised double-blind clinical trial
Source: Trials. 2025 Oct 17;26:422. doi: 10.1186/s13063-025-09099-9 (PMC12535095; doi:10.1186/s13063-025-09099-9)

Supplemental Digital Content

This appendix has been provided by the authors to provide additional information

**CRYSTALLBrain: Crystalloid Fluid Choice and Neurological Outcome in Patients with non-traumatic Subarachnoid Haemorrhage – a study protocol for a multi-centre randomised double-blind clinical trial**

Anna S. Messmer, Matthieu Pitteloud, Hervé Quintard, Urs Pietsch, Martin Müller, Miodrag Filipovic, Stephan M. Jakob, Werner J. Z’Graggen, Joerg C. Schefold, Carmen A. Pfortmueller

Table of content

[Figure S1. Modified Rankin Scale (mRS) 3](#_Toc188033808)

[Figure S2. Glasgow Outcome Scale Extended (GOSE) 3](#_Toc188033809)

[Figure S3. Mini MOCA 3](#_Toc188033810)

## Figure S1. Modified Rankin Scale (mRS)

| **Level** | **Description** |
| --- | --- |
| 0 | No symptoms |
| 1 | No significant disability, despite symptoms; able to perform all usual duties and activities |
| 2 | Slight disability; unable to performed previous activities but able to look after own affairs without assistance |
| 3 | Moderate disability; requires some help, but able to walk without assistance |
| 4 | Moderately severe disability; unable to walk without assistance and unable to attend to own bodily needs without assistance |
| 5 | Severe disability; bedridden, incontinent and requires nursing care and attention |

## Figure S2. Glasgow Outcome Scale Extended (GOSE)

| Scale | Interpretation |
| --- | --- |
| 1 = dead | Dead |
| 2 = vegetative state | Absence of awareness of self and environment |
| 3 = lower severe disability | Needs full assistance in ADL* |
| 4 = upper severe disability | Needs partial assistance in ADL |
| 5 = lower moderate disability | Independent, but cannot resume work/school or all previous social activities |
| 6 = upper moderate disability | Some disability exists, but can partly resume work or previous activities |
| 7 = lower good recovery | Minor physical or mental deficits that affects daily life |
| 8 = upper good recovery | Full recovery or minor symptoms that do not affect daily life |

*ADL = activities of daily life

## Figure S3. Mini MOCA


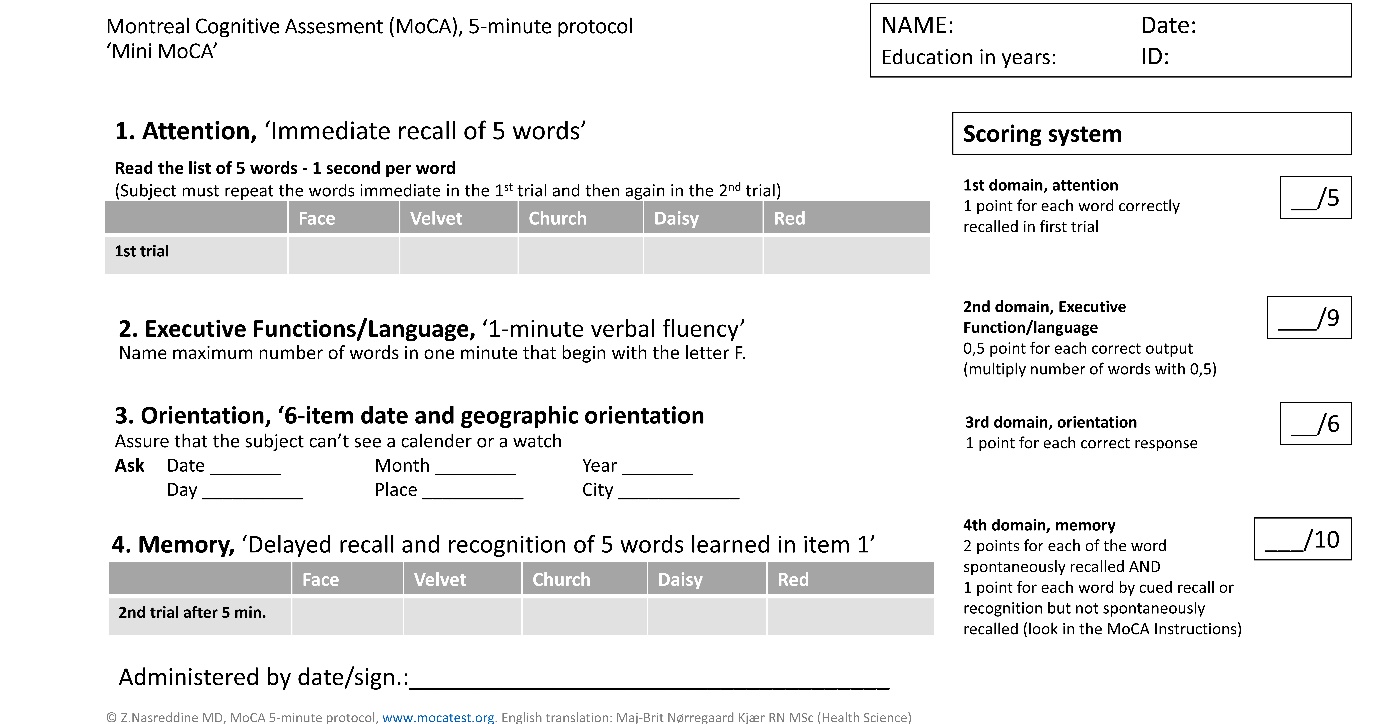

Supplement: Supplementary file 1 — Supplementary material 1. [file 13063_2025_9099_MOESM1_ESM.docx]
